# Electrochemistry Properties of Fullerene C60-Retinol Nanostructured Electrodes Synthesized by Electrochemical Methods

**DOI:** 10.1021/acsomega.5c10039

**Published:** 2026-02-03

**Authors:** Fatma Bayrakçeken Nişancı

**Affiliations:** Faculty of Science, Department of Chemistry, 37503 Ataturk University, 25240 Erzurum, Turkey

## Abstract

In this paper, the controlled nanometer-scale growth of Fullerene C60, Retinol, and Fullerene C60-Retinol has been successfully achieved using electrochemical methods. In particular, the Fullerene C60–Retinol hybrid increased the number of active electrochemical sites by facilitating electron transfer to oxygen species adsorbed on the material surface. This finding is supported by results obtained using EIS and several other characterization methods. We demonstrate that fullerene C60-Retinol nanostructured thin-film electrodes exhibit a significant reduction in charge transfer resistance compared to their pure counterparts, and that incorporating C60 into retinol can be highly effective in reducing the charge transfer barrier during electrolytic processes. In light of these results, the electrochemically synthesized C60–retinol hybrid materials can be considered promising for future integration into biomedical-related technologies, provided that their biocompatibility and stability are verified in subsequent studies.

## Graphical abstract

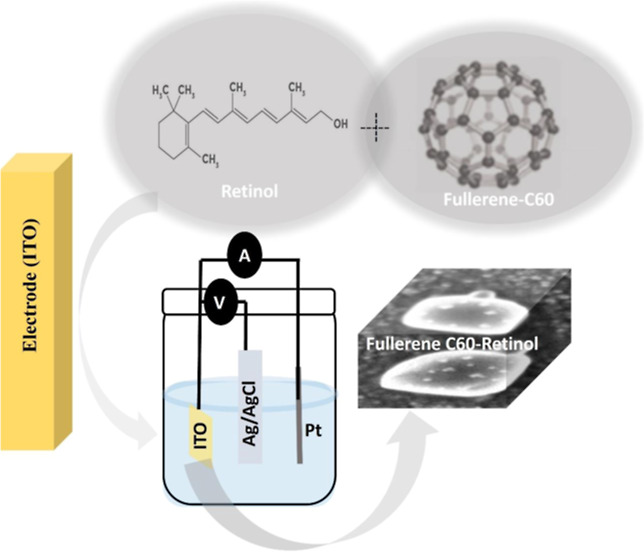

## 1 Introduction

Fullerene-C60 is utilized in a wide range of applications due to its unique chemical and physical properties, conductivity, and ease of chemical modification.^1–3^ These applications include biomedical fields (e.g., gene and drug delivery)^4^ as well as nonbiomedical fields (e.g., supercapacitors, hydrogen storage, and nanoelectronics).^5–12^ Current demands for reliable electrochemical biosensors necessitate the development of sensors with large surface areas to allow molecular modification, good biocompatibility to maintain biological activity, and excellent conductivity for electron transport.^13^ In this context, fullerene-C60 has the ability to be easily modified by functional groups, high carrier capacity, biocompatibility, a relatively wide potential range and electrochemical activity for various redox reactions.^14,15^ This has brought about their widespread use in recent years as both electrode modifiers and nanocarriers in the preparation of electrochemical preparations.^16–18^ Moreover, they are free from metallic impurities, relatively easy to apply, and lead to reproducible electrochemical reactions.^19^ Their unique dimensional and electronic structures^20,21^ make them attractive mediators in electrochemical biosensors. Fullerenes are a promising family of electroactive compounds with rich electrochemistry. They enable operation at lower potentials, thus reducing interferences from electroactive compounds.^22,23^ Fullerene structure is similar to graphite, consisting of 60 carbon-like ball-like pentagons and hexagons. However, while graphite has only six-membered rings in its structure, fullerenes can have five-membered rings and are used in cosmetic preparations as a high-quality, antiaging raw material with excellent biological activity.^24^ Fullerenes have great potential in the pharmaceutical sector and also have an important place in the field of nanomedicine.^25^ With their excellent antioxidant and antibacterial properties, water-soluble fullerenes have been shown to be effective against AIDS (behave as a strong antioxidant due to the large amount of double bonds they contain).^26–31^ The biggest challenge for scientists was the insolubility of these molecules in water and their tendency to form aggregates. This problem can be overcome by using techniques such as encapsulating fullerenes with hydrophilic molecules, suspending them in other solvents, and conjugating them with hydrophilic molecules. These modifications enhance their ability to penetrate cells, reach the nuclei and mitochondria, and scavenge intracellular free radicals. In addition, its ability to cross the blood–brain barrier thanks to its small size makes many medical applications possible in the nanomedical field in terms of developing new active compounds that can be used by the brain. Pharmacokinetic studies over the last 25 years have shown that dissolved C60 is absorbed by the gastrointestinal tract and excreted within a few hours, so its toxicity is very low. Therefore, its use has been suggested for the encapsulation of certain drugs (in biomedical applications such as cancer treatments and neurodegenerative diseases).^32^

Similarly to C60, retinol exhibits several attractive properties, including high electronic conductivity, large surface area, good biocompatibility, chemical inertness, structural stability, and strong adsorption capacity to organic molecules, and plays many roles in human metabolism. The main benefits of retinol are reducing fine lines and wrinkles, improving sun spots and skin texture irregularities, treating acne, pimples, melasma and other hyperpigmentation issues. Retinol increases skin cell production, helps unclog pores and removes dead skin cells, and increases collagen production, reducing the appearance of fine lines and wrinkles, which are signs of aging.^33,34^ Retinol is a compound with multifunctional effects on photodamaged skin, including the production of hyaluronic acid, collagen, and elastin, as well as epidermal proliferation and differentiation.^35–38^ Retinol, when applied to the skin, penetrates the cells and turns into retinoic acid. At this point, retinoic acid is effective in accelerating the renewal process of skin cells. Thus, it prepares the ground for the emergence of new and healthy cells. Retinoic acid is widely used to prevent skin aging and to treat psoriasis.^39^ It also helps in the immune system and in the regulation of cell growth, embryonic stem cell differentiation and development, and in maintaining the healthy structure and function of the skin and all epithelial tissues of the body.^40,41^

C60 Fullerene and Retinol have been synthesized using techniques such as hydrothermal techniques,^42–45^ physicochemical deposition^46–48^ ball milling,^49,50^ nanoclustering.^51–54^ These techniques have some disadvantages such as requiring very high vacuum systems that are quite expensive and difficult to maintain, interfacial diffusion of atoms at the interface due to the need to work at very high temperatures, difficulty in their application, and requiring devices consisting of complex and expensive parts. Electrochemical-based techniques seem to be able to overcome such problems. An electrochemical process is important in that it involves low-cost equipment, homogeneous coating can be achieved regardless of the shape of the substrate used, and does not involve an experimental process that will cause little harm to the environment.^55^

In conclusion, the synergistic properties of Fullerene C60 and Retinol highlight the potential of their electrochemical synthesis to provide reliable, practical, and sustainable solutions, especially in the development of functional materials and electrochemical devices. This study reports the first example of the controlled electrochemical deposition of a C60–retinol hybrid, demonstrating a simple and effective route for fabricating uniform hybrid films with tunable morphology and strong interfacial adhesion on ITO substrates. The research specifically focused on measuring and comparing the electrochemical behavior, surface morphology, structural properties, and optical characteristics of pure C60, pure Retinol, and their hybrid nanostructures. The experimental work involved the controlled electrodeposition of C60 and Retinol to form hybrid thin films, followed by comprehensive analyses using cyclic voltammetry (CV), electrochemical impedance spectroscopy (EIS), open-circuit potential (OCP), UV–Vis spectroscopy, Raman spectroscopy, and X-ray diffraction (XRD). The direction of the research is oriented toward understanding the interfacial interactions, charge-transfer mechanisms, and synergistic effects between the organic (Retinol) and carbon-based (C60) components. The ultimate goal of this study is to develop a stable, conductive, and morphologically homogeneous hybrid nanomaterial with potential applications in electrochemical sensors and bioelectronic devices.

## 2 Experimental Section

A BAS 100B/W electrochemical workstation engaged to a three-electrode cell (C3 Cell Stand, BAS) was used for the electrochemical procedures. The powder X-ray diffractograms of the deposited nanostructures were enrolled using a Rigaku powder X-ray diffractometer with a CuK-X-ray source (λ = 1.5406̊A). The morphological analysis and elemental composition determination Retinol, Fullerene C60 and Fullerene C60-Retinol nanostructures were carried out by a ZEISS system coupled to the scanning electron microscope and Hitachi High Technology HT7700 brand transmission electron microscope (TEM).

Molecular absorption studies were carried out using Shimadzu brand UV-3101PC model spectrophotometer and WITech alpha 300R brand Micro Raman Spectrometer to obtain information about the bonds formed by Retinol, Fullerene C60 and Fullerene C60-Retinol nanostructured electrodes. Electrochemical measurements were implemented in a three-electrode electrochemical cell configuration using the Retinol, Fullerene C60 and Fullerene C60-Retinol nanostructures deposited on ITO as a working electrode. Figure 1 schematically illustrates the stepwise electrochemical synthesis and formation mechanism of the nanostructured ITO/Fullerene C60/Retinol hybrid electrode. In the first stage, Fullerene C60 nanoparticles are electrochemically deposited onto the ITO substrate, forming a homogeneous and conductive thin film that acts as a nucleation platform. In the subsequent step, Retinol molecules are electrodeposited onto the Fullerene-modified surface, where π–π stacking interactions and hydrogen bonding between Retinol and the π-conjugated C60 surface promote uniform nucleation and the development of a stable hybrid architecture. This process leads to the intimate integration of the two components, resulting in improved electron transport pathways and enhanced electrochemical activity. The schematic representation is significant as it visually summarizes the sequential deposition process, the interfacial interactions between Fullerene C60 and Retinol, and the formation of the hierarchical nanostructure responsible for the superior electrochemical performance demonstrated in this study (Figure 1).

**Figure 1:**
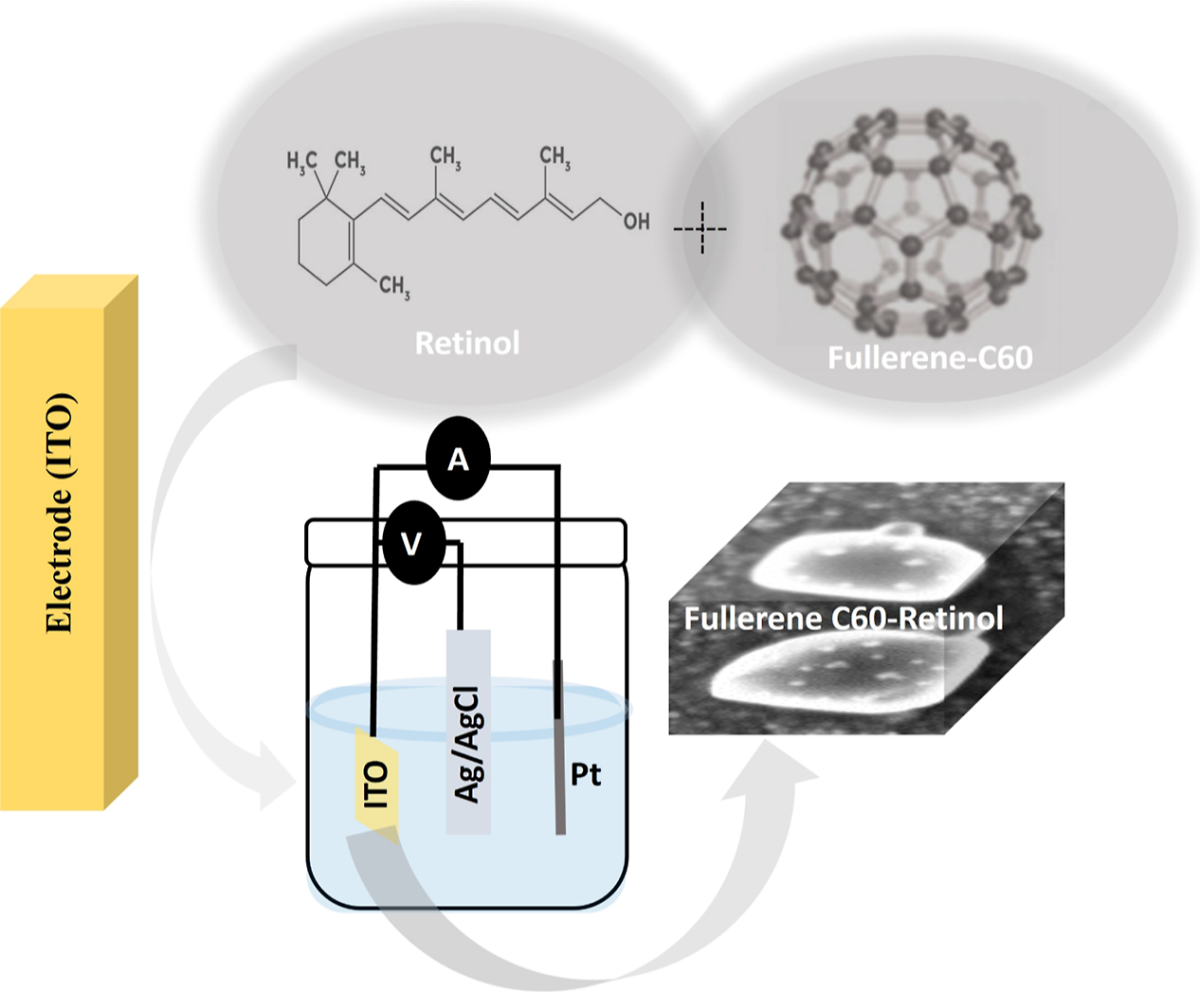
Electrochemical synthesis of ITO/Fullerene C60/Retinol nanostructured arrays and schematic representation.

## 3 Results and Discussion

Voltammograms of the deposition regions of Retinol and Fullerene C60 are shown on the same axis in Figure 2. Retinol thin film electrodes were synthesized in a solution containing 0.1 M KCl, 0.03 g Retinol, pH: 5 acetate buffer at a constant potential of −0.40 V at 25 °C for 60 min. Fullerene C60 thin film electrodes; in 0.01 g fullerene C60, 0.1 M (TBA)­BF_4_/MeCN­(1:1), the coating process was carried out at −0.75 V at 25 °C for 60 min electrodeposition time. As shown in the voltammograms in Figure 2, the electrochemical behavior of Retinol was investigated at 25 °C on ITO electrodes in a solution containing 0.1 M KCl, 0.03 g Retinol, and acetate buffer at pH 5. When the electrode potential was scanned from +1.40 V toward cathodic potentials, a cathodic peak appeared at approximately −0.40 V, corresponding to the deposition of Retinol. During the reverse anodic scan from −0.60 V, two anodic peaks were observed at approximately +0.50 V and +0.89 V, which are attributed to the stripping (oxidative dissolution) of the deposited Retinol layers. Based on these observations, a potential of −0.40 V was selected as the electrodeposition potential for Retinol in subsequent experiments.

**Figure 2:**
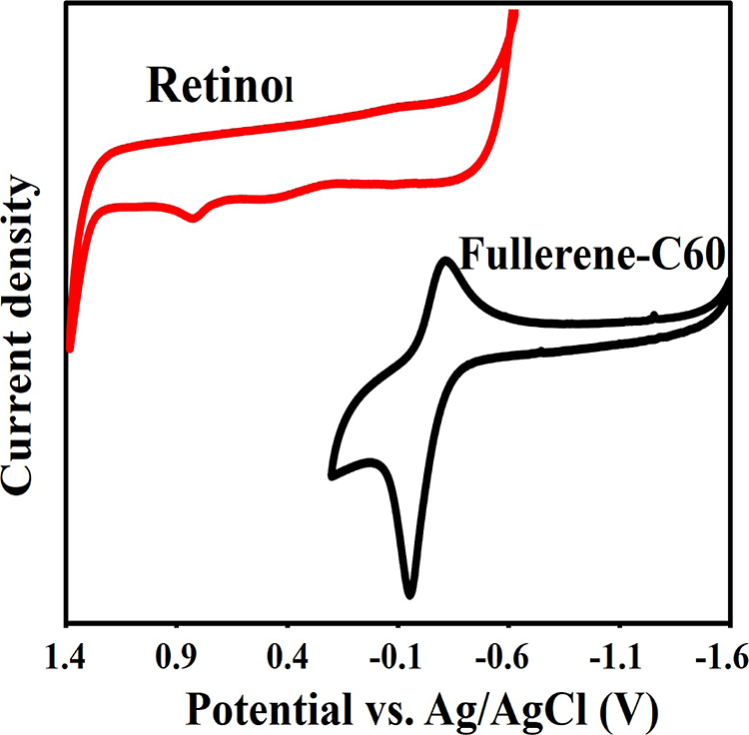
Cyclic voltammetry (CV) curve of the Retinol and Fullerene samples measured in a solution containing 0.1 M KCI, 0.03 g Retinol, pH:5 acetate buffer 25 °C at a scan rate of 20 mV s^–1^ and 0.01 g fullerene C60, 0.1 M (TBA)­BF_4_/MeCN­(1:1).

At room temperature, the electrochemical behavior of Fullerene C60 was examined on ITO electrodes using a solution containing 0.01 g Fullerene C60 and 0.1 M (TBA)­BF_4_ in MeCN (1:1, v/v). When the potential was scanned from +0.20 V toward cathodic values, a cathodic peak appeared at approximately −0.60 V, corresponding to the reduction and deposition of Fullerene C60. Upon the reverse scan up to +1.60 V, an anodic peak was observed at approximately −0.25 V, indicating the desorption (oxidative removal) of the deposited Fullerene C60 layer. Well-organized thin films of Fullerene C60 were obtained when the electrode potential was held constant at −0.75 V, within the range of −0.80 V to −0.65 V (Figure 2).

Figure 3 shows the SEM images and EDS data obtained as a result of electrodeposition on ITO electrodes in order to examine the formation of Retinol, Fullerene C60, and Fullerene C60-Retinol thin films. In addition, these data are important to determine the catalytically active sites, composition and structure of Fullerene C60, Retinol, Fullerene C60-Retinol thin film electrodes.^56–61^ Homogeneously distributed 3D prismatic Retinol nanoparticles are clearly seen in the SEM images of Retinol obtained as a result of 15 min of electrodeposition at room temperature onto ITO electrodes at −0.40 V. Fullerene C60 was deposited at room temperature for 10 min at −0.75 V, and retinol was deposited on this fullerene thin film for 5 min at −0.40 V. In SEM images of the resulting Fullerene C60-Retinol heterostructures, modified Retinol particles were clearly observed on the 3D polyhedral particles (Figure 3).

**Figure 3:**
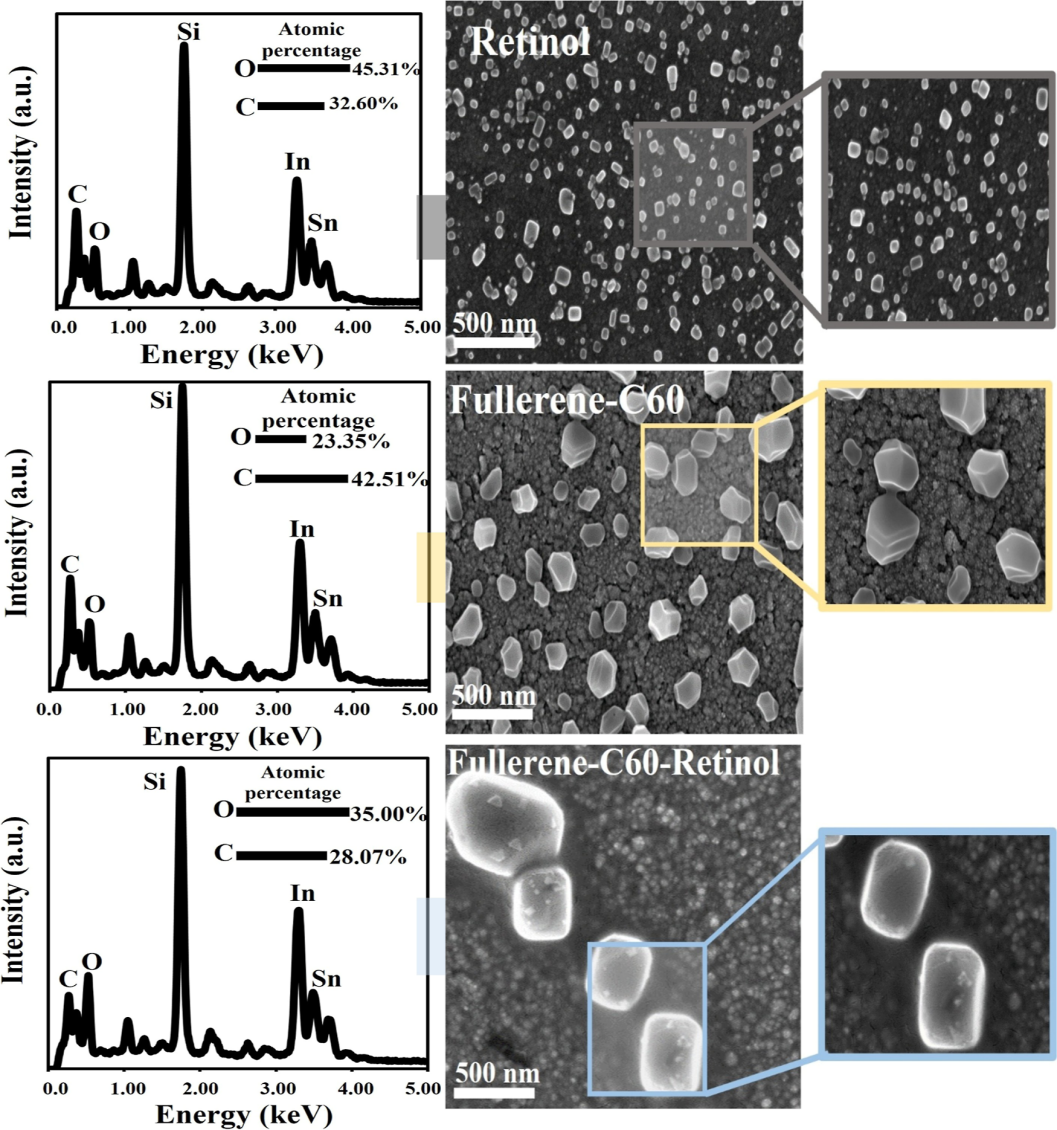
FE-SEM images and energy dispersive spectroscopy (EDS) data of the electrochemically synthesized Retinol, Fullerene C60 and Fullerene C60-Retinol nanoparticles.

The Fullerene C60–Retinol electrode exhibited the largest surface area, as evidenced by its porous and homogeneously distributed structure in the SEM images. After the electrochemical deposition process, interfacial interactions occur between retinol molecules and the π-conjugated surface of fullerene C60. These interactions promote nucleation and the subsequent growth of retinol around the fullerene particles. As a result, the fullerene acts as a nucleation center, facilitating the aggregation and formation of larger and more compact retinol structures on its surface. Meanwhile, the fullerene particles become embedded or homogeneously dispersed within the retinol and appear smaller in the SEM images due to partial surface coverage (Figure 3). This behavior indicates a strong interfacial interaction and synergistic assembly between the two components. In this context, the increased surface area imparts the Fullerene C60–Retinol electrode with an enhanced current response, reduced charge-transfer resistance, and superior electrochemical performance, primarily arising from the synergistic electronic interactions between Fullerene C60 and Retinol.

The surface morphologies of the Retinol, Fullerene–C60, and Fullerene C60–Retinol films were examined by SEM (Table 1). The Retinol film exhibited small, uniformly distributed prismatic structures with an average size of 55 ± 10 nm. In contrast, the Fullerene–C60 film consisted of larger, polyhedral-shaped particles with an average size of 115 ± 25 nm. Upon electrochemical codeposition, the Fullerene C60–Retinol hybrid displayed well-formed prismatic structures with an average particle size of approximately 135 ± 20 nm. The increase in particle size and improved uniformity indicate a strong interfacial interaction between C60 and Retinol, resulting in more compact and homogeneous film morphology. The EDS spectra of Fullerene C60-Retinol films show that the percentage of oxygen element content increases when compared to Fullerene C60 alone and Retinol alone. In this case, it is clearly shown that the increase in the amount of O originates from the Retinol content modified by electrodeposition on the fullerene C60 (Figure 3).

**Table 1:** Average Particle Sizes and Morphological Characteristics from SEM Images

sample	average size (nm)	morphological description
Retinol	55 ± 10	small, uniformly distributed prismatic structures
Fullerene C60	115 ± 25	larger polyhedral particles with sharp edges
Fullerene C60-Retinol	135 ± 20	well-defined prismatic hybrid structures with uniformity

In Raman spectroscopy of fullerene C60 (Figure 4a), the band at 1370 cm^–1^ is also evident, the ID band and the higher wavenumber IG band at 1591 cm^–1^. The G band at 1591.7 cm^–1^ is the first-order spectrum due to bond stretching of sp^2^ carbon atoms in both cyclic and chained flat structures. Also, the D bands are indicative of some structural disorders such as frames and amorphous carbon species.^62–67^ In Raman spectra (Figure 4a), the main bands of Retinol are located in the spectral ranges of 1000–1660 cm^–1^ and the most intense band corresponding to stretching (C=C) is at 1591 cm^–1^. Other less intense bands at 1456 cm^–1^ are assigned to CH_2_ exchange.^68^ Compared with the Fullerene C60-Retinol spectrum in Figure 4a, the pure retinol and pure Fullerene C60 spectra showed an increase in Raman scattering intensity.^69,70^ That is, the band at 1591 cm^–1^ can be clearly distinguished. The similar Raman spectra of Fullerene C60, Retinol and Fullerene C60-Retinol electrodes indicate that the carbon phase of the investigated electrodes consists of a carbon structure in which differently hybridized carbon atoms are mixed together. The reason for the very slight shifts in the band values, especially in the low intensity spectra, is the functional groups formed in the carbon layers.

**Figure 4:**
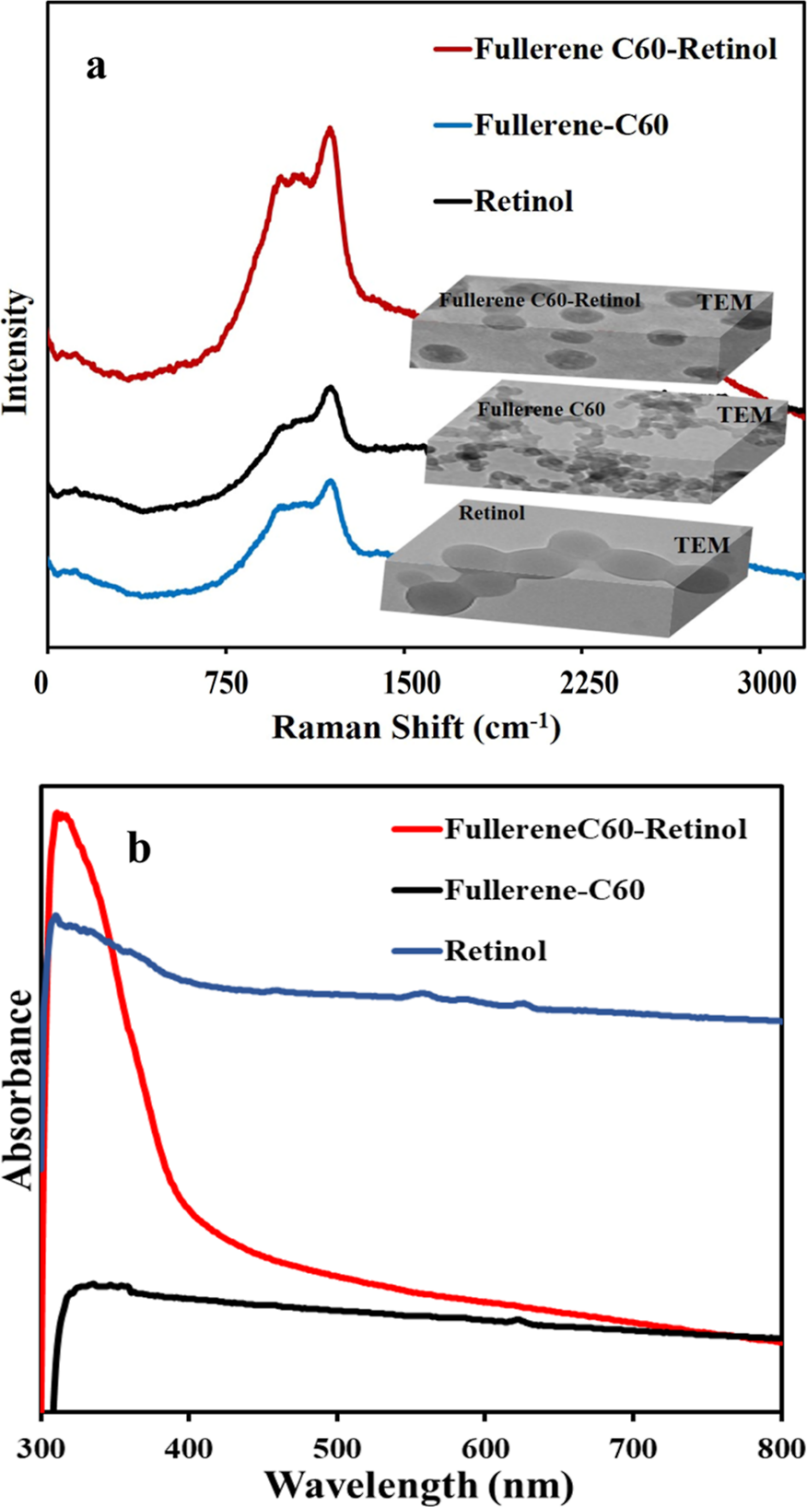
(a) Raman spectra and TEM of Retinol, Fullerene C60, Fullerene C60-Retinol and (b) UV–visible absorption spectra of the electrochemically synthesized Retinol, Fullerene C60 and Fullerene C60-Retinol nanoparticles.

Figure 4b shows the UV–vis absorbance spectra of Fullerene C60-Retinol, Fullerene C60-Retinol. As can be seen, the maximum absorbance intensity of Fullerene C60-Retinol was observed at approximately 321 nm. This is different from the peaks of pure fullerene C60 and pure Retinol in that the spectrum is a single spectrum that is sharper than the peaks of pure Fullerene C60 and pure Retinol. This situation is attributed to the effects of fullerene C60 molecules on Retinol in the UV–vis absorption spectra. The appearance of a new and slightly red-shifted absorption peak in the UV–vis spectrum of the Fullerene C60–Retinol hybrid structure indicates the formation of strong electronic interactions between the π-conjugated system of Fullerene C60 and the conjugated double bonds of Retinol. This spectral shift can be attributed to interfacial interactions between the two components. These interactions result in a new electronic transition state that is distinct from those of pure Fullerene C60 or pure Retinol. The red shift and increased absorption intensity suggest enhanced electron delocalization within the hybrid nanostructure, confirming the successful formation of a stable Fullerene C60–Retinol complex. The small spectra at approximately 310 and 368 nm in the UV–vis spectra of pure Retinol and pure Fullerene C60 are thought to be hydrated by the electrolyte solution during electrodeposition.^71–74^ When examined individually, C60 (2.45 eV) and Retinol (2.82 eV) each exhibit their characteristic energy band gaps. However, upon electrochemical combination into a hybrid structure, strong interfacial interactions form between C60 and Retinol, facilitating charge transfer and orbital overlap. These interactions facilitate charge transfer and orbital overlap between C60 and Retinol, causing their molecular orbitals to approach each other and form new intermediate electronic states. This new configuration reduces the energy required for electronic transitions, resulting in a narrower band gap (2.35 eV). Consequently, the decrease in band gap indicates that electrons in the hybrid system can be more easily excited, supporting enhanced electrical conductivity and more efficient charge transfer within the hybrid electrode. The formation of Fullerene C60-Retinol nanostructures enhanced by UV–vis data was confirmed consistent with the findings of previous SEM, TEM, Raman studies.

In order to better understand the Fullerene C60, Retinol, Fullerene C60-Retinol structures obtained from SEM images, their crystal structures were elucidated by X-ray diffraction XRD with CuKα radiation (Figure 5). The diffraction peaks occurring at 2θ = 21.25 correspond to the fullerene-C60 hexagonal prism (311) structure, while 2θ = 25.1 corresponds to the diffraction peaks of 3D cubic retinol and the other peaks originate from the ITO substrate. In Fullerene C60-retinol, it is clearly observed that the intensity of the (311) diffraction peak belonging to Fullerene C60 decreases and in retinol-coated Fullerene C60, the intensity of the diffraction peak belonging to retinol increases. Fullerene C60 was excellently modified with retinol by electrochemical deposition and its synergistic activity was supported by XRD, SEM studies.^75,76^

**Figure 5:**
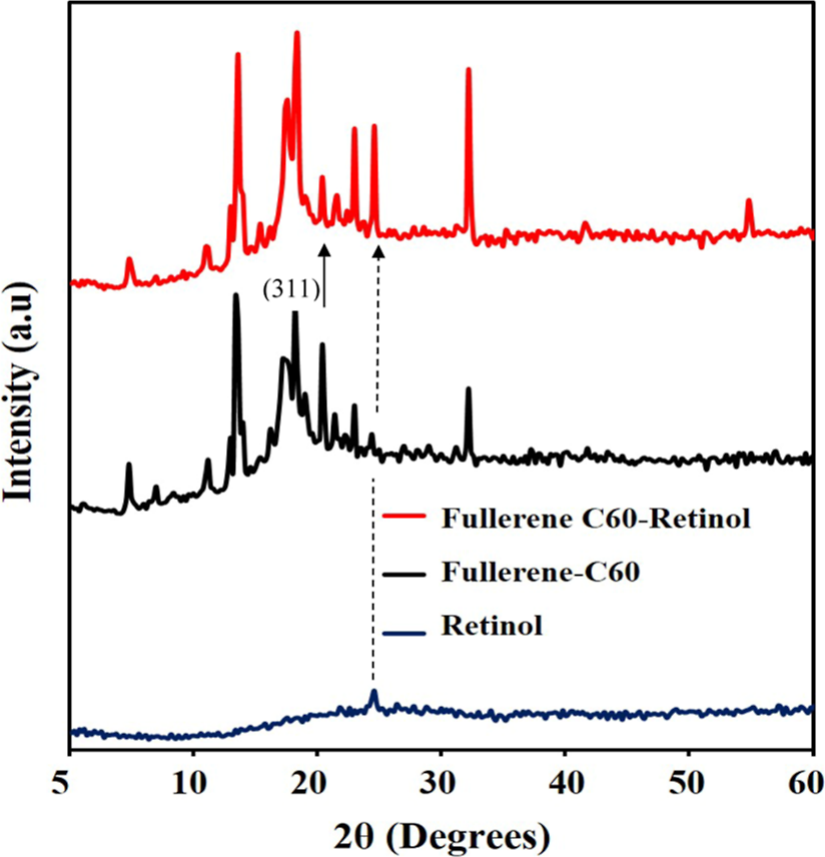
X-ray Diffraction (XRD) data of Retinol, Fullerene-C60 and Fullerene C60-Retinol at different film thicknesses. Radiation source: CuKα.

Electrochemical analysis (Figure 6) obtained for electrode prepared on ITO electrode in 0.1 M KCl solution containing 10 mM Fe­(CN)_6_^3–^/Fe­(CN)_6_^4–^. CV data of Fullerene C60, Retinol and Fullerene C60-Retinol electrodes electrochemically coated on ITO electrodes, taken at different scan rates (from 10 mV/s to 70 mV/s) for each of them in 0.1 M KCl electrolyte containing 10 mM Fe­(CN)_6_^3–^/Fe­(CN)_6_^4–^ at room temperature are shown in Figure 6. In the CV data, an increase in the current density of the anodic peaks with increasing scan rate and a large number of active areas of the surface at different scan speeds was observed.

**Figure 6:**
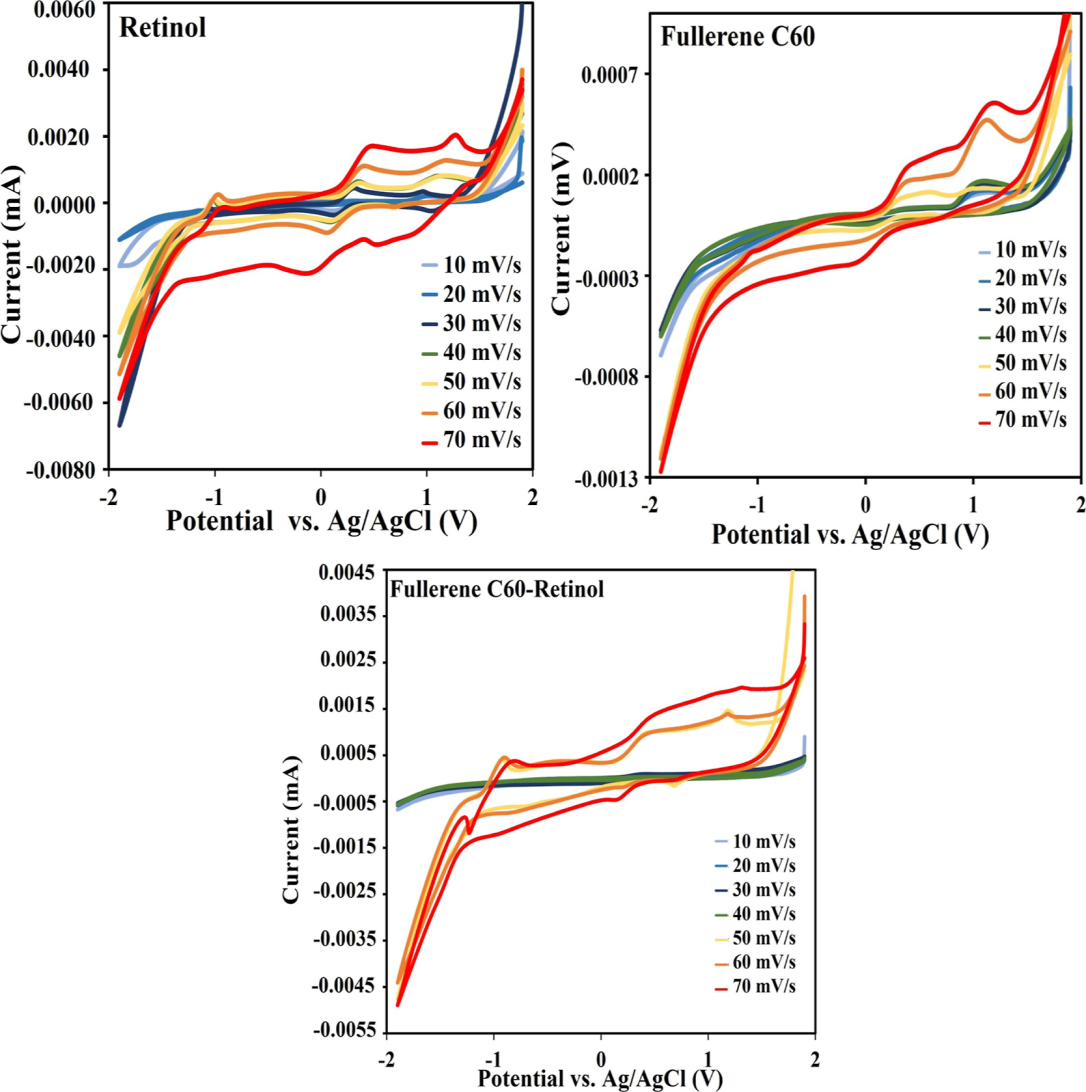
Cyclic voltammetry (CV) of the Retinol, Fullerene C60 and Fullerene C60-Retinol electrode performed at scan rates ranging from 10 to 70 mV/s.

Especially in fullerene C60-retinol electrodes, as a result of the interaction between two different materials, the amount of carbon element increases and higher current values occur with the increase in surface activity. In the CV tests performed over multiple consecutive cycles at scan rates ranging from 10 to 70 mV/s in 0.1 M KCl containing 10 mM Fe­(CN)_6_^3–^/Fe­(CN)_6_^4–^, the electrode maintained a nearly constant anodic and cathodic peak current, indicating excellent reproducibility and minimal degradation of electroactive sites. Repeated cyclic voltammetry (CV) measurements show that the electrode maintains its current response almost unchanged after several consecutive cycles, indicating high stability and reproducibility.

The enhanced cyclic voltammetry (CV) performance of the Fullerene C60–Retinol electrode can be attributed to a donor–acceptor driven interfacial charge-transfer mechanism established within the hybrid structure (Figure 6). Owing to its low-lying LUMO energy levels and high electron affinity, Fullerene C60 functions as an efficient electron acceptor and conductive transport network, whereas retinol, containing conjugated C = C bonds and oxygen-containing functional groups, exhibits electron-donating behavior under electrochemical polarization. During electrochemical deposition, strong interfacial interactions such as π–π stacking and hydrogen bonding facilitate the uniform nucleation and growth of retinol on the C_60_-modified surface, resulting in a homogeneous hybrid film with an increased electroactive surface area. The resulting electronic coupling enables partial charge transfer from retinol to C_60_, effectively lowering the interfacial charge-transfer barrier and accelerating redox kinetics. Consequently, the hybrid electrode displays higher current densities, improved electrochemical reversibility, and reduced charge-transfer resistance. Moreover, the systematic increase in anodic and cathodic peak currents with increasing scan rate confirms fast and predominantly surface-controlled electron-transfer behavior.^77–79^

To further investigate the electrochemical properties of all samples, EIS test was performed (Figure 7a). Samples were immersed in a solution of 0.1 M KCl electrolyte containing 10 mM Fe­(CN)_6_^3–^/Fe­(CN)_6_^4–^ at 25 °C. Then, Nyquist plots of the samples are shown in Figure 7a. Electrochemical addition of C60 to Retinol decreases the charge transfer resistance and charge transfer barrier at the interface between the catalyst and the electrolyte, thus increasing the electrochemical reaction activity of the electrode. The catalytic performance of the tested material shows superiority compared to pure Retinol and pure Fullerene C60, Figure 7a. The diameter of the capacitive semicircle is related to the charge transfer resistance.^80^ EIS results provide information about the charge transfer resistance (Rct) and ion transport properties of the electrode–electrolyte interface. In the Nyquist plots, the diameter of the semicircle corresponds to the Rct value. A smaller semicircle indicates lower charge transfer resistance, suggesting enhanced electron transfer and improved surface conductivity.

**Figure 7:**
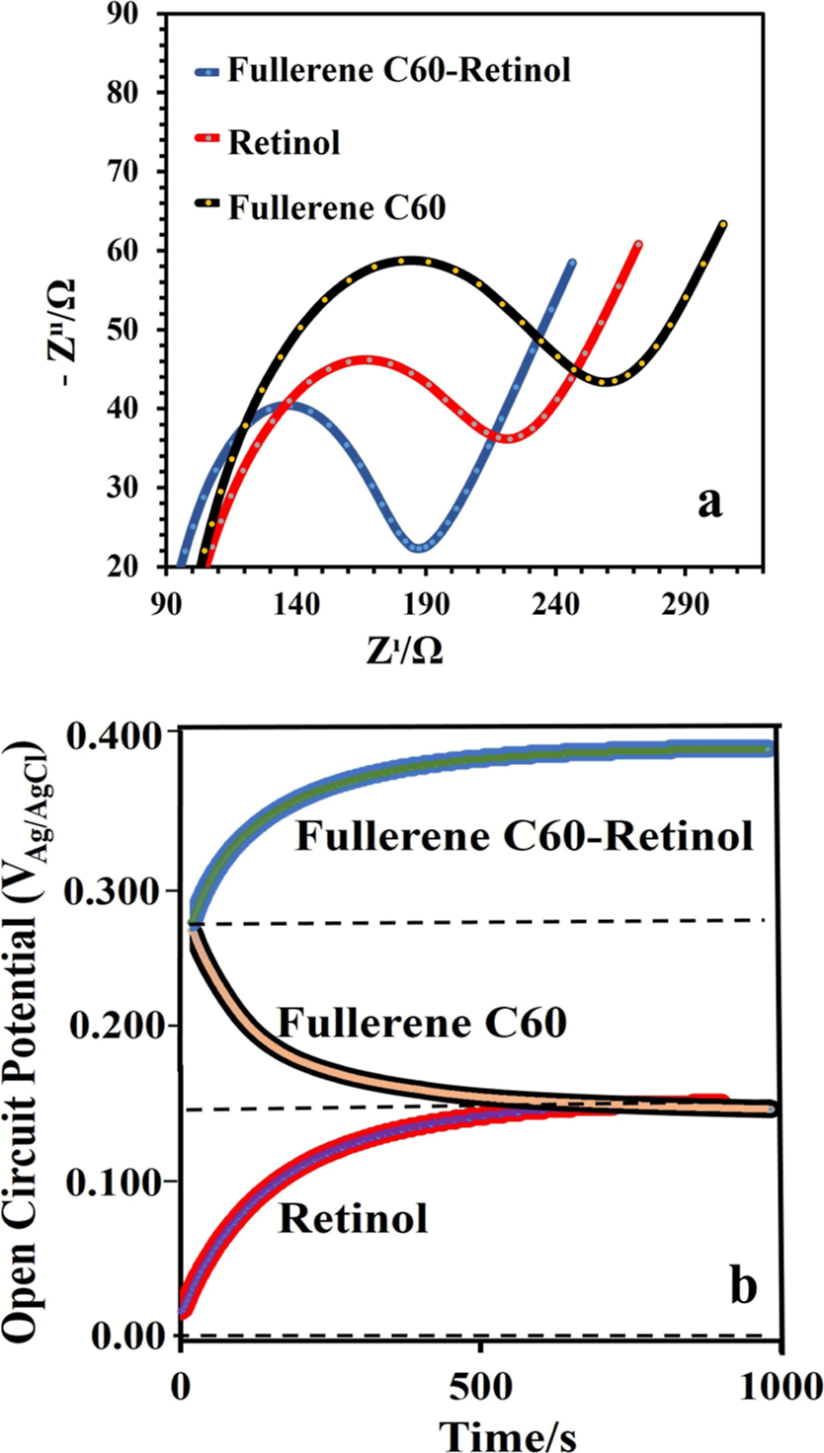
Electrochemical tests: impedance, cyclic voltammetry, and open circuit potential (OCP) curves data. (a) Electrochemical impedance spectra (EIS) in Nyquist plot of the Retinol, Fullerene C60 and Fullerene C60-Retinol electrode in 10 mM K_3_Fe­(CN)_6_, 10 mM K_4_Fe­(CN)_6_ and 0.1 M KCl. Frequency range: 0.1–105 Hz. (b) Open circuit potential (OCP) curves, E­(OCP)/time plots for Retinol, Fullerene C60 and Fullerene C60-Retinol samples in 10 mM K_3_Fe­(CN)_6_, 10 mM K_4_Fe­(CN)_6_ and 0.1 M KCl.

The significant decrease in *R*_ct_ observed for the Fullerene C60–Retinol electrode compared to pure C60 and pure Retinol confirms that the combination of these two materials synergistically facilitates electron transfer and increases electrochemical activity. Figure 7b shows the variation of OCP of Fullerene C60, Retinol and Fullerene C60-Retinol electrodes with immersion time in 0.1 M KCl electrolyte containing 10 mM Fe­(CN)_6_^3–^/Fe­(CN)_6_^4–^ at room temperature. For the Fullerene C60-Retinol electrodes, only 400 s were required for the OCP to gradually reach the highest OCP value and then remain constant. However, the OCP of pure Fullerene C60 shifts to lower positive potentials for the samples, while the pure Retinol electrode exhibits higher OCP than pure fullerene C60 and takes relatively longer to reach the OCP potential constant.^81,82^ OCP measurements, on the other hand, indicate the equilibrium potential between the electrode and the electrolyte in the absence of an applied current. The more positive and stable potential observed for the Fullerene C60–Retinol electrode reflects higher electron density and improved surface stability. Furthermore, the shorter time required to reach a steady-state potential demonstrates that the Fullerene C60–Retinol surface exhibits superior electrical conductivity and electrochemical stability compared to its individual components. The EIS results are consistent with the open circuit potential results. Overall, the combined EIS and OCP analyses demonstrate that the electrochemically synthesized Fullerene C60–Retinol electrodes possess enhanced electron transfer kinetics, higher stability, and improved conductivity, confirming their strong potential for applications in electrochemical and biomedical systems.^83,84^

## 4 Conclusions

In this study, C60–Retinol hybrid films were successfully electrochemically synthesized on ITO electrodes for the first time. SEM analysis revealed that the average particle sizes were approximately 40–80 nm for Retinol, 80–150 nm for Fullerene C60, and 100–180 nm for the C60–Retinol hybrid, confirming the formation of larger and more uniform prismatic nanostructures due to the synergistic interaction between Retinol and C60. The optical band gap estimated from UV–Vis spectra decreased from 2.45 eV (C60) and 2.82 eV (Retinol) to 2.35 eV for the C60–Retinol hybrid, supporting improved electronic coupling between the two components. Electrochemical analyses (CV, EIS, and OCP) revealed a remarkable improvement in charge transfer efficiency, reduced interfacial resistance, and enhanced surface stability for the C60–Retinol hybrid electrode compared to the individual components. Open-circuit potential and cyclic voltammetry measurements indicated stable electrochemical behavior with consistent current responses over repeated cycles, highlighting the electrode’s durability and reproducibility. Overall, the electrochemically synthesized C60–Retinol hybrid electrode exhibits pronounced synergistic effects between the organic and carbon-based components, offering a promising platform for advanced electrochemical sensors and bioelectronic devices. These results provide fundamental insight into hybrid nanostructure formation and highlight their potential for developing next-generation functional materials with superior electrochemical and structural performance.

## Data Availability

All data generated or analyzed during this study are included in this published article.
